# Ectopic parotid gland in the left cheek: a case report

**DOI:** 10.1186/s13256-023-03862-9

**Published:** 2023-04-14

**Authors:** Soheila Borji, Pegah Moharrami Yeganeh

**Affiliations:** 1 Department of Radiology, Vali-E-Asr Hospital, School of Medicine, Zanjan University of Medical Sciences, Zanjan, Iran; 2 School of Medicine, Zanjan University of Medical Sciences, Zanjan, Iran

**Keywords:** Ectopic parotid, Parotid gland, Magnetic resonance imaging, MRI, Case report

## Abstract

**Background:**

The parotid glands are one of the major components of the salivary glands. Their function is to secrete serous saliva to facilitate chewing and swallowing. The normal position of the parotid glands is anterior to and below the lower half of the ear; superficial, posterior, and deep to the ramus of the mandible.

**Case presentation:**

In this article, we present a rare case of an ectopic left parotid gland located in the left cheek of a 45-year-old Middle-Eastern female who presented with a painless mass inside the left side of her face. Magnetic resonance imaging revealed a well-defined mass in the left buccal fat, which was isosignal with the right parotid gland.

**Conclusion:**

Further evaluations of detected cases are necessary to obtain more information regarding the pathogenesis and possible etiologies of this condition. There is a need for more reports of similar cases, as well as diagnostic and etiologic studies, to understand the cause of this condition further.

## Introduction

The parotid glands are one of the major components of the salivary glands. The normal position of the parotid glands is anterior to and below the lower half of the ear; superficial, posterior, and deep to the ramus of the mandible. They are surrounded by the mastoid bone and the sternocleidomastoid muscle posteriorly, the parotid fascia and the skin laterally, and the masseter muscle anteriorly, and extend to the parapharyngeal space medially [1].

Ectopia of the salivary system is defined by the presence of the salivary system components, whether a complete organ or nonorganized tissue, in a location other than their normal anatomic positions. This condition is a rare abnormality that has been reported very few times. In this review, we present a case of an ectopic parotid gland in a 45-year-old patient.

## Case presentation

A 45-year-old Middle-Eastern woman with no significant medical history presented to the clinic complaining of the presence of a painless mass inside the left side of her face, recently discovered.

On physical examination, an approximately 2 × 1 cm painless mass was palpated in the left cheek, which was not visible externally. No skin changes or signs of inflammation were present. She stated that she had not noticed the mass in her face before.

**Fig. 1:**
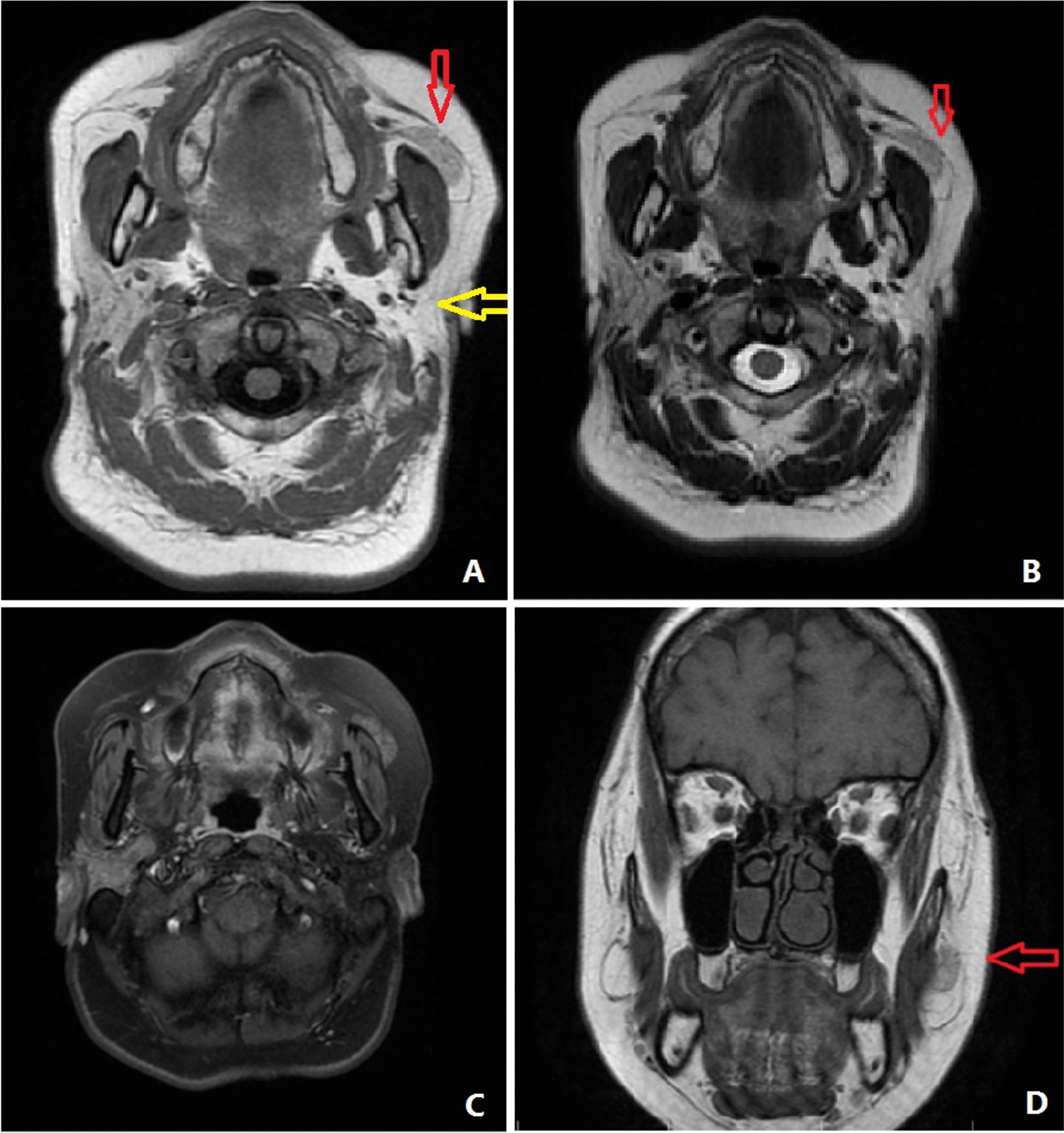
**A** Axial T1 fluid attenuated inversion recovery (FLAIR) sequence MRI. The left parotid gland is not seen in the normal anatomic area (yellow arrow) and located in left buccal fat (red arrow). **B** Axial T2-weighted MRI. The left parotid gland is not seen in the normal anatomic area and is located in left buccal fat (red arrow). **C** Axial T1 fat-saturated with gadolinium. The same mass with enhancement similar to the right parotid gland. **D** Coronal T1-weighted image, with the mass in left buccal fat (red arrow)

Magnetic resonance imaging (MRI) revealed an isosignal to high signal well-defined mass measuring 23 × 10 mm in the left buccal fat, which was isosignal with the right parotid gland. The left parotid gland was not detected in the normal anatomic site (Fig. 1).

Based on the clinical and MRI findings, the diagnosis of ectopic parotid gland was considered for the patient. Since the patient had no other complaints regarding mastication or any other oral conditions and no complications were detected, she was dismissed with no further procedures as she did not consent to further investigations.

## Discussion and conclusion

Ectopic parotid gland is an extremely rare condition, with very few reports in the literature. More common cases of parotid ectopia include accessory glands and heterotopic gland tissue. The accessory gland is defined by the presence of an ectopic gland with a duct system, which is distinct from the main parotid gland, while heterotopic gland tissue is the presence of ectopic parotid tissue in other locations, without a duct system [2]. The accessory parotid gland is usually located adjacent to, but separate from the main gland [2].

The most common reported locations for ectopic salivary gland tissue are the cervical lymph nodes [3], the middle ear [4] and periauricular area [5], the bones of maxilla and mandible [6, 7], and the soft tissues of the neck and mouth [8, 9]. It has also been reported in association with some congenital anomalies and syndromes [7, 10].

Our case presents a woman in her mid-40s, with a mass that brought no complaints except for the mere physical presence of the mass, and no complications. Interestingly, most of the previously reported cases involved patients of a young age [4, 7, 10–12], as expected in most congenital conditions; whereas in our case, the patient has lived through four decades without developing anything requiring medical attention. Moreover, reported cases are usually associated with complications such as fistulous ducts or malignant changes [9, 12, 13], while our patient’s clinical and radiologic findings showed no signs of such complications, and thus removed the need for any further tests or evaluation. Also, nearly all of the reviewed cases discussed the ecptoia of salivary tissue or an “accessory” gland, with the main gland present in its normal location. There was one report that described cases of the main parotid gland’s ectopia similar to our case [7], but those cases were in young individuals associated with congenital anomalies of the maxillary region, which did not exist in our case. Another report of a 12-year-old female with a diagnosis of bilateral ectopic parotid glands was found, without coexisting anomalies [14].

Supposedly, these congenital abnormalities form within the period of fetal development. During embryogenesis, parotid glands begin to form in the sixth gestational week, from a tunnel-like structure within the mesenchyme of the primitive mouth, originating from ectoderm [15]. These invaginations are close to the adjacent lymphatic tissue [16]. We hypothesize that it might be possible for these invaginations to then move alongside the lymphatic tissue, and later be placed and ultimately develop into the parotid glands in an ectopic locus. Further evaluation of future detected cases is necessary to obtain more information regarding this condition’s pathogenesis and possible etiologies. Most reported cases of parotid ectopia were associated with various congenital anomalies. Still, there were also reported cases of ectopia without coexisting conditions, as well as our case, suggesting that there might be other causes of parotid ectopia. There is a need for more reports of similar cases, as well as diagnostic and etiologic studies, to understand the cause of this condition further.

## Data Availability

Not applicable.
